# miR-744-5p contributes to ocular inflammation in patients with primary Sjogrens Syndrome

**DOI:** 10.1038/s41598-020-64422-5

**Published:** 2020-05-04

**Authors:** Qistina Pilson, Siobhan Smith, Caroline A. Jefferies, Joan Ní Gabhann-Dromgoole, Conor C. Murphy

**Affiliations:** 1 School of Pharmacy and Biomolecular Sciences (PBS) and RSCI Research Institute, Royal College of Surgeons in Ireland, Dublin 2, Ireland; 2 Department of Ophthalmology, Royal College of Surgeons in Ireland, Dublin 2, Ireland; 3 Department of Ophthalmology, Royal Victoria Eye and Ear Hospital, Dublin 2, Ireland; 4 Division of Rheumatology, Department of Medicine, Cedars-Sinai Medical Centre, 8700 Beverly Blvd, Los Angeles, California, 90048 USA; 5 Department of Biomedical Sciences, Cedars-Sinai Medical Centre, 8700 Beverly Blvd, Los Angeles, California, 90048 USA

**Keywords:** Epigenetics, Autoimmunity, Chronic inflammation

## Abstract

In primary Sjögren’s syndrome (pSS) the exocrine glands become infiltrated with lymphocytes instigating severe damage to the salivary and lacrimal glands causing dry eyes and dry mouth. Previous investigations have suggested that dysregulated localized and systemic inflammation contributes to the development and pathogenesis of pSS. A miR microarray performed in primary human conjunctival epithelial cells (PECs) demonstrated significant differences in miR expression at the ocular surface between pSS patients and healthy controls. MicroRNA-744-5p (miR-744-5p) was identified as being of particular interest, as its top predicted target is Pellino3 (PELI3), a known negative regulator of inflammation. Validation studies confirmed that miR-744-5p expression is significantly increased in PECs from pSS patients, whilst PELI3 was significantly reduced. We validated the miR-744 binding site in the 3’ untranslated region (UTR) of PELI3 and demonstrated that increasing PELI3 levels with a miR-744-5p antagomir in an inflammatory environment resulted in reduced levels of IFN dependent chemokines Rantes (CCL5) and CXCL10. These results reveal a novel role for miR-744-5p in mediating ocular inflammation via Pellino3 expression in pSS patients and suggest that miR-744-5p may be a potential therapeutic target for the management of severe dry eye disease and ocular inflammation in pSS patients.

## Introduction

Sjögren’s syndrome (SS) is a systemic autoimmune disorder characterized by dry eyes and dry mouth secondary to reduced exocrine function of both the lacrimal and salivary glands^1^. Due to the impaired exocrine gland function, dryness can extend to other parts of the body such as the skin, lungs and vaginal tract. SS occurs either as a primary condition (pSS) or as a complication in individuals with other inflammatory disorders including rheumatoid arthritis (RA) and systemic lupus erythematosus (SLE) where it is termed secondary Sjögren’s syndrome. pSS has a general incidence of approximately 0.5-3% of the population. It can occur at any age but is most common between the ages of 40 and 60 years, with women 9 times more likely to suffer from SS than men^2–4^. Chronic inflammation, accompanied by increased lymphocytic infiltration of exocrine glands, is the pathological hallmark of this disease. Aqueous deficient dry eye disease (DED) in patients with SS is caused by a failure of the lacrimal glands to secrete tears^5,6^.

Increased levels of inflammatory cytokines including interleukin (IL)-6, IL-12, tumour necrosis factor alpha (TNF-α)^7–9^ and more recently IL-23^10^ have been observed both locally and systemically and have been shown to play an important role in SS pathogenesis^11–13^. Autoantibodies such as anti-SSA/Ro and anti-SSB/La are a characteristic hallmark of SS and are thought to contribute to pathogenesis through the formation of immune complexes and associated inflammation. More recently autoantibodies targeting the muscarinic receptor type III (M3R) have been shown to alter membrane trafficking of aquaporin 5 (AQP5), a protein involved in transmembrane water transport, in salivary glands of SS patients thus contributing to impaired fluid secretion^14^.

In striving to understand what drives the tissue specific and systemic inflammation associated with SS, focus has shifted to investigating the contribution of microRNAs (miRNAs or miRs) to the pathogenesis of pSS^15–17^. miRs are tiny fragments of 18-25 non coding base pairs playing a posttranslational role, regulating approximately 90% of protein-coding genes, and play a central role in various biological processes including immune cell lineage commitment, differentiation, proliferation, apoptosis and maintenance of immune homeostasis^18^. Dysregulated expression of noncoding RNAs, including miRs, has been associated with immunopathology of inflammatory autoimmune conditions such as systemic lupus erythematosus (SLE)^19^. Studies in an American cohort of SS patients demonstrated increased salivary gland expression of miR-155, a known regulator of inflammation, in patients with reduced salivary flow^17^. Indeed many of the miRs whose expression is altered in pSS are key regulators of inflammation and cytokine signalling^15,17,20–22^. For example, miR-125, miR-155 and miR-378 are all increased in SS and promote inflammation by enhancing pro-inflammatory cytokine signalling or by attenuating anti-inflammatory processes. Principally they target negative regulators of inflammation including Src homology 2 (SH2) domain-containing inositol-5ʹ-phosphatase 1 (SHIP1) and suppressor of cytokine signalling 1 (SOCS1). Other miRs including miR-9, miR-21, miR-146, miR-147 and miR-187 are decreased in SS and attenuate inflammation by repressing positive regulators of inflammation including programmed cell death protein 4 (PDCD4), nuclear factor kappa-light-chain-enhancer of activated B cells (NFκB), interleukin-1 receptor-associated kinase 1 (IRAK1) and TNF receptor associated factor 6 **(**TRAF6)^20,22^. It has been suggested that inflammation and exocrine gland destruction in SS involves a complicated interplay between cytokine networks, innate immune cells and their mediators^23–27^. There is now strong evidence to suggest that alterations in miR expression contribute to the initiation and progression of pSS, although a functional link to pathogenic cytokine production has yet to be established.

While there is significant interest in pSS and the area of DED and an acceptance that inflammation is the key driving factor, the role of miRs in the pathogenesis of inflammation of the ocular surface has not been explored. Specifically in the context of SS patients, who present with severe DED the majority of miR studies have focused on animal models, peripheral blood mononuclear cells and salivary gland biopsies^15–17,28–30^. In order to fully investigate events at the ocular surface we optimised the isolation of miR from conjunctival epithelial cells (CECs) by impression cytology^31^. Using this technique we have demonstrated significant differences in miR expression at the ocular surface between pSS patients and healthy controls. MicroRNA-744-5p (miR-744-5p) was identified as being of particular interest, as its top predicted target is Pellino3 (PELI3), a member of the Pellino E3 ubiquitin ligase family, is a known negative regulator of inflammation^32^. PELI3 plays an important physiological role by negatively regulating TLR3 signaling via suppression of TRAF6-mediated polyubiquitination of interferon regulatory factor 7 (IRF7), blocking its activity and expression of interferon beta (IFN-β)^33^. Validation of this miR and its predicted gene target confirmed that miR-744-5p expression is significantly increased in CECs from pSS patients, whilst its predicted gene PELI3 was significantly reduced. Furthermore manipulation of miR-744-5p expression using a mimic or antagomir resulted in reduced and increased expression of PELI3, respectively. This study validated the miR-744 binding site in the 3′ untranslated region (UTR) of PELI3 and demonstrated that increasing PELI3 levels with a miR-744-5p antagomir in an inflammatory environment resulted in reduced levels of IFN dependent chemokines Rantes (C-C motif) ligand 5 (CCL5)) and C-X-C motif chemokine 10 (CXCL10). Overall these studies reveal a novel role for miR-744-5p in mediating ocular inflammation via Pellino3 expression in SS patients.

## Results

### Altered miRNA expression at the ocular surface in pSS patients

**Figure 1:**
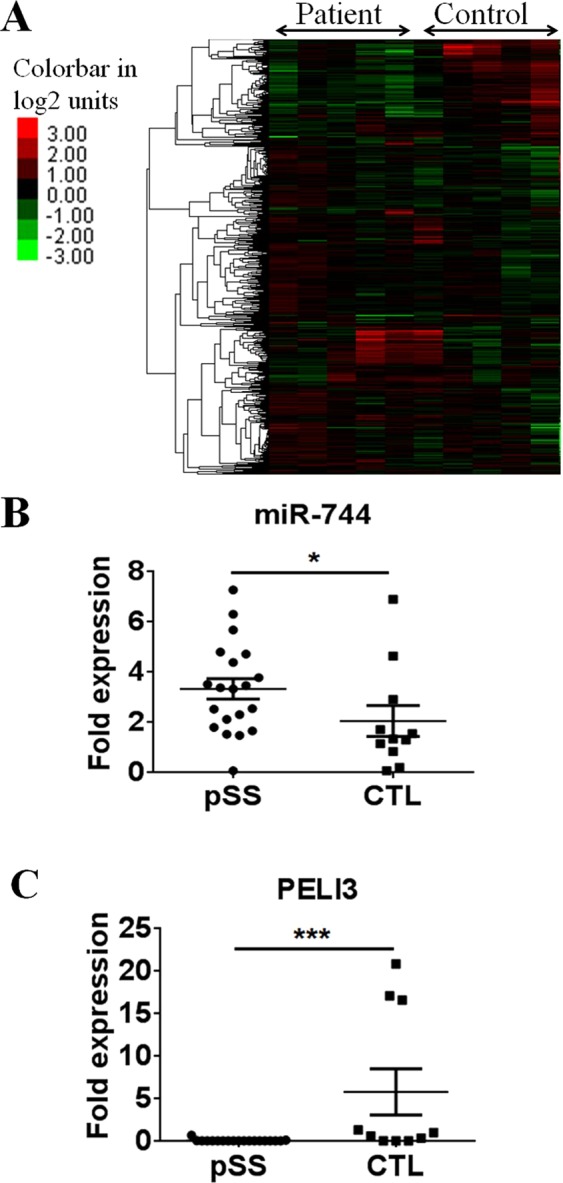
Altered expression of microRNA-744-5p (miR-744-5p) and its target gene *Pellino3* in conjunctival epithelial cells from patients with primary Sjogrens Syndrome (pSS) as compared with normal controls. (**A**) Microarray analysis of miRNA expression in primary conjunctival epithelial cells from patients with pSS and healthy controls. (**A**) Heatmap displaying relative expression of miRs in pSS patients compared to healthy controls (n = 5). (**B**) Independent verification in conjunctival epithelial cells of *hsa-miR-744-5p* expression in 19 pSS patients and 11 healthy controls (CTL) by quantitative real-time polymerase chain reaction (qPCR) analysis, ^**^P ≤ 0.01. (**C**) Conjunctival epithelial cell expression of *Pellino3* in 19 pSS patients and 11 healthy controls, as determined by qPCR analysis. Each data point represents a single subject; horizontal lines show the mean, ^*^P ≤ 0.02 ^***^P ≤ 0.001.

Previous investigations have focused on salivary gland or peripheral blood mononuclear cells in human and mice studies of pSS. More recent studies have examined the levels of inflammatory cytokines and chemokines in ocular washes from pSS patients^34–37^. Consistent with these investigations we observed significantly enhanced production of interferon gamma (IFNγ), IL-2, IL-4, IL-5, IL-10, IL-12, IL-13 and TNFα (Supplemental Fig. 1). To more fully address the unmet need to understand events occurring at the ocular surface our initial study sought to identify potential differentially expressed miRNA in primary human conjunctival cells (PECs) isolated from SS patients that may be contributing to ocular surface inflammation. A total of 20 patients with pSS who fulfilled the AECG criteria were included in this study. Eleven healthy volunteers constituted the control group. Patient demographic data and results of ocular surface parameters are presented in Table 1. The expression of >2000 miRNAs in samples derived from 5 pSS patients and 5 healthy controls by impression cytology (IC) were investigated by Ocean Ridge Bioscience using ORB MirBASE Version 19 MicroRNA Microarray. This study revealed differential expression profiles of miRNA in patients with pSS as compared with healthy controls (Fig. 1A). Among these miR-744-5p was of interest due to previous reports showing altered expression in the autoimmune condition SLE^38,39^ as well as potentially functioning as a regulator of transforming growth factor beta 1 (TGF-β1) synthesis^40^. Additionally, by targeting the ubiquitously expressed phosphatase protein tyrosine phosphatase 1B (PTP1B), miR-744-5p has been shown to play a feedforward role in the type 1 interferon (IFN) pathway by positively enhancing the expression of IFN induced genes (CCL2, CCL5, CXCL10 and IL6)^41^. Significantly increased expression of miR-744-5p (P ≤ 0.02) was observed in a larger cohort of PECs derived from 19 patients with pSS and 11 healthy controls (Fig. 1B). Comparing multiple miRNA target prediction programs including, miRDB, miRWalk and DIANA, we identified Pellino 3 (PELI3) as a putative target of miR-744-5p and demonstrated significantly reduced (P ≤ 0.01) expression of the gene in PECs from pSS patients compared to healthy controls (Fig. 1C).

**Table 1:** Demographics and results of ocular surface parameters of patients. Data shown are number and percentage of patients under each American European Consensus group classification criteria (AECG) category.

Patient Characteristic	AECG
	(n = 20)
Positive diagnosis (%)	20 (100)
Mean age in years (Range)	57.88 (35-70)
Female (%)	15 (75)
Male (%)	5 (25)
Disease duration (Years)	5.18 (0.17-28)
Symptoms	
Dry eyes (%)	20 (100)
Dry mouth (%)	20 (100)
**Systemic involvement (%)**	15 (75)
Constitutional (%)	4 (20)
Lymphadenopathy (%)	3 (15)
Glandular (%)	1 (5)
Articular (%)	7 (35)
Cutaneous (%)	1 (5)
Respiratory (%)	6 (30)
Renal (%)	0
Muscular (%)	1 (5)
PNS (%)	0
CNS (%)	0
Haematological (%)	5 (25)
Biological (%)	13 (65)
**Irish (%)**	20 (100)
Schirmer’s 1 (mm/5 min)	1.75 ± 2.5
TBUT (seconds)	2.5 ± 0.88

TBUT: tear film breakup time.

### Modulation of miR-744-5p expression in primary human conjunctival cells

**Figure 2:**
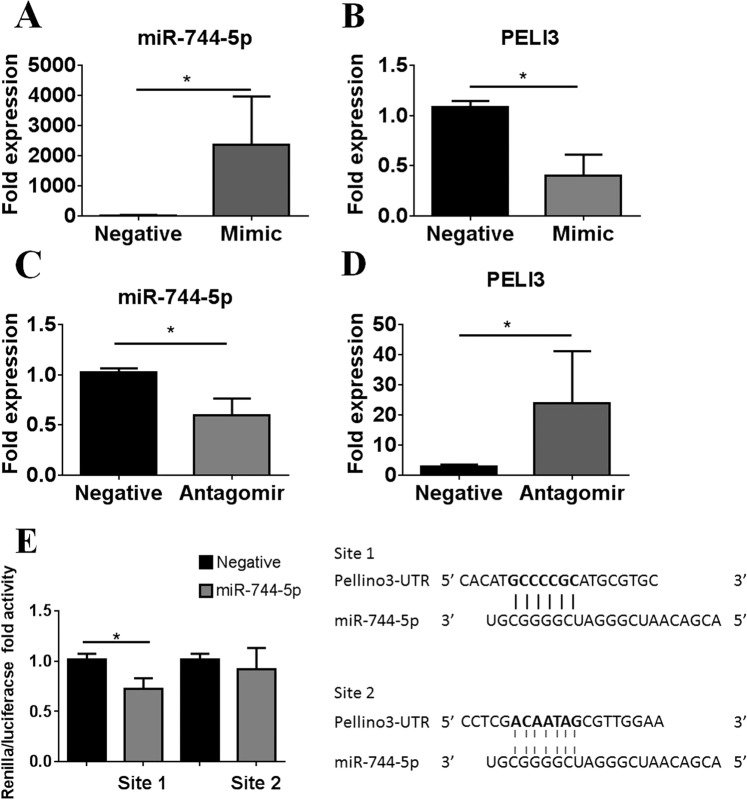
Modulation of miR-744-5p expression in primary human conjunctival cells. (**A**,**B**) Expression of *miR-744-5p* and Pellino3 (PELI3) in miR-744-5p mimic transfected primary human conjunctival epithelial cells, isolated from healthy volunteers by impression cytology, after 72 h as determined by real-time PCR. Values are the mean ± SD of 4 samples, ^*^P ≤ 0.02. (**C**,**D**) Expression of *miR-744-5p* and Pellino3 (PELI3) in miR-744-5p antagomir transfected primary human conjunctival epithelial cells, isolated from healthy volunteers by impression cytology, after 72 h as determined by real-time PCR. Values are the mean ± SD of 4 samples, ^*^P ≤ 0.02. (**E**) Luciferase activity in HEK293T cells transfected with the 3′UTR of Pellino3 containing the miR-744-5p binding site (Site 1) and an unrelated fragment control (Site 2) and either 50 nm of negative control or miR-744-5p mimic. Values are the mean ± SD of 4 samples, ^*^P ≤ 0.02.

To establish PELI3 as a genuine target of miR-744-5p we transfected PECs derived from healthy controls with a miR-744-5p mimic which demonstrated significantly enhanced expression of miR-744-5p and significantly reduced (*P* ≤ 0.02) PELI3 expression (Fig. 2A,B). Transfection with a miR-744-5p antagomir resulted in significantly reduced miR-744-5p expression and significantly enhanced PELI3 expression (Fig. 2C,D). To determine if the effects of miR-744-5p on PELI3 expression were direct, we cloned 2 regions of the PELI3 3′UTR into a luciferase reporter construct, one containing the conserved putative miR-744-5p binding site (Site 1), as well as a region from the PELI3 3′UTR that contained no miR-744-5p binding site, the unrelated fragment control (Site 2). Co-transfection of HEK293T cells with the two reporter constructs with either the miR-744-5p mimic or negative control demonstrated that transfection of HEK293T cells with a miR-744-5p mimic significantly attenuated Site 1 luciferase activity but failed to inhibit activity of the Site 2 luciferase construct (Fig. 2E), indicating that miR-744-5p directly targets the 3′ UTR of PELI3. Taken together, our data demonstrates that PELI3 is both a novel and direct target of miR-744-5p.

### Poly I:C mediated inflammation is reduced in human conjunctival cells treated with a miR-744-5p antagomir *ex vivo*

To determine the potential relevance of modulating PELI3 expression in pSS, we examined the possibility of enhancing PELI3 expression under inflammatory conditions using a miR-744-5p antagomir in primary human conjunctival epithelial cells (HConEC) which were sourced from Innoprot. To induce an inflammatory environment that is comparable to that observed in pSS patients, HConECs were treated with polyinosinic:polycytidylic acid (Poly(I:C)). Previous studies in female NZB/WF1 SS prone mice have shown that Poly(I:C) treatment resulted in accelerated development of salivary gland disease which was associated with the production type I IFN, inflammatory cytokines and chemokines^42,43^. Treatment of healthy mice with TLR3 ligand has been shown to result in increased expression of proinflammatory cytokines in salivary glands and lacrimal tissue from healthy mice^44–46^. Investigations in murine conjunctival epithelium cells have found these cells respond to Poly(I:C) treatment and have suggested that TLR3 plays a critical role in regulating ocular surface inflammation^47^. Furthermore, human conjunctival epithelial cells have been shown to express TLR3 and produce pro-inflammatory cytokines including IL-6, IL-8, CXCL10, CXCL11, Rantes and MCP-1 following Poly(I:C) treatment^48,49^.

**Figure 3:**
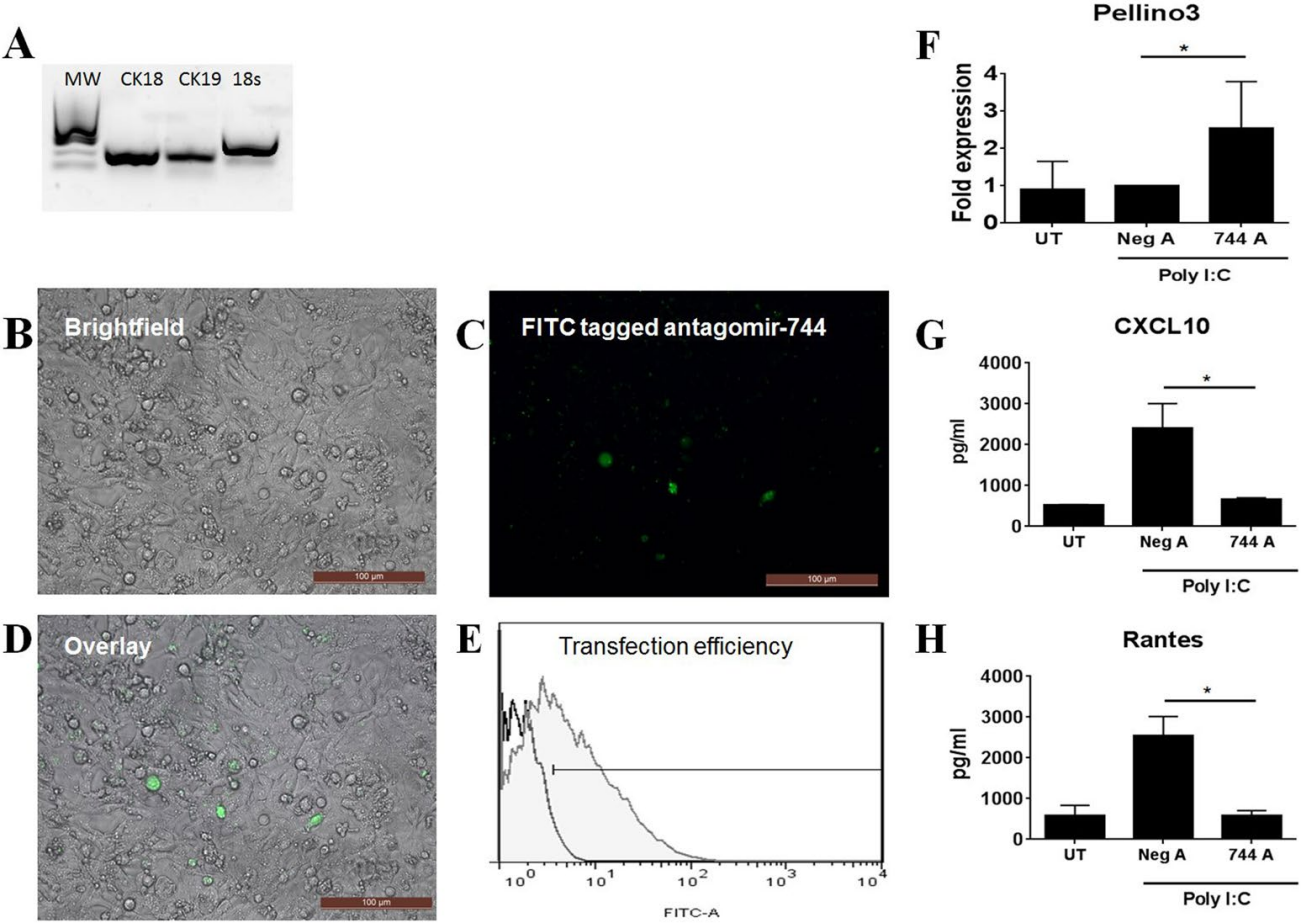
Poly(I:C) mediated inflammation is reduced in human conjunctival cells treated with a miR-744-5p antagomir *ex vivo*. (**A**) PCR analysis of cytokeratin-18 (CK18) and cytokeratin-19 (CK19) in primary human conjunctival epithelial cells (HConEC) from Innoprot. Results are representative of 3 samples, with 18 s used as a control. The full gel image is included in the supplementary data. (**B**–**E**) Human conjunctival epithelial cells (Innoprot) were transfected with a FITC-labeled miR-744-5p antagomir for 72 hours. (**B**–**D**) Transfection of miR-744-5p antagomir was visualised using an inverted bright field microscope (Leica, DMIL) x20 magnification. (**E**) Uptake FITC-labelled miR-744-5p antagomir assessed by flow cytometry after 72 h of culture (filled histogram) relative to negative antagomir-treated cells (unfilled histogram). Data are representative of three independent experiments. (**F**–**H**) Human conjunctival epithelial cells (Innoprot) were treated with a miR-744-5p antagomir for 72 hours. Cells were lither left untreated or cultured with polyinosinic:polycytidylic acid (Poly I:C) during the final 24 hours of culture. (**F**) Expression of *Pellino3* in miR-744-5p antagomir transfected human conjunctival epithelial cells (Innoprot) after 72 hours was determined by real-time PCR. Values are the mean ± SD of 6 samples, ^*^P ≤ 0.03. (**G**,**H**) Levels of CXCL10 and Rantes in human conjunctival epithelial cells (Innoprot) as determined by ELISA. Values are the mean ± SD of 3 samples, ^*^P ≤ 0.03, ^**^P ≤ 0.005. UT = untransfected cells, Neg A = negative antagomir and 744A = miR-744-5p antagomir.

Firstly, we characterised HConECs by determining expression of the epithelial specific markers cytokeratin-18 (CK18) and cytokeratin-19 (CK19) by PCR (Fig. 3A). Secondly we examined transfection of HConECs with a FITC-labelled miR-744-5p antagomir (Exiqon) using an inverted bright field microscope (Fig. 3B–E) and quantified this uptake by flow cytometry after 72 h of culture. These studies demonstrated significant transfection of miR-744-5p in HConECs (filled histogram) relative to negative antagomir-treated cells (unfilled histogram) (57.33 ± 5.03, *P* ≤ 0.003) (Fig. 3E). Finally, HConECs were either left untreated or cultured with Poly(I:C) during the final 24 hours of antagomir transfection. Significantly increased expression of PELI3 in these Poly(I:C) cells was accompanied by a significant reduction (*P* ≤ 0.03) in Rantes and CXCL10 levels relative to negative antagomir-treated cells (Fig. 3F–H). Our data demonstrates that increasing PELI3 expression represents a mechanism to modulate inflammation at the ocular surface.

## Discussion

SS is a complex autoimmune disease with multifactorial pathogenesis and multisystem manifestations. There is no cure for SS and current treatments aim to alleviate disease symptoms by treating dry eye with artificial tears and using anti-inflammatories to treat localised and systemic inflammation. In many patients these treatments show little long term clinical benefit due to problems with maintaining therapeutic concentrations of the agents at the ocular surface and unwanted side effects. In the context of SS-related DED prolonged use of immunosuppressive agents such as corticosteroids and cyclosporine can result in glaucoma, cataracts and an increased susceptibility to ocular infections^50^. In addition, systemic administration of biologic and immunosuppressive therapies do not help to ameliorate ocular surface inflammation in SS related dry eye disease, indicating the need to explore other inflammatory pathways to control this disease^51–53^. Our studies reveal a novel role for miR-744-5p in mediating ocular surface inflammation via modulating PELI3 expression and reducing inflammation in primary human conjunctival cells.

MiR-744-5p has shown utility as a plasma biomarker for pancreatic cancer^54^, in addition to being expressed in the plasma of patients with wet age-related macular degeneration^55^. In the context of autoimmunity previous reports have demonstrated reduced expression of this miRNA in the naive B cell subset of SLE patients as well as increased expression of miR-744 in peripheral blood mononuclear cells from patients with lupus nephritis compared with healthy controls^38,39^. Finally miR-744 transfection in HK-2 cells was shown to inhibit endogenous TGF-β1 synthesis which has important implications for inflammation^40^.

We observed significantly enhanced expression of this miRNA in PECs derived from pSS patients and identify PELI3 as a novel target, whose expression was significantly reduced in PECs derived from pSS patients.

**Figure 4:**
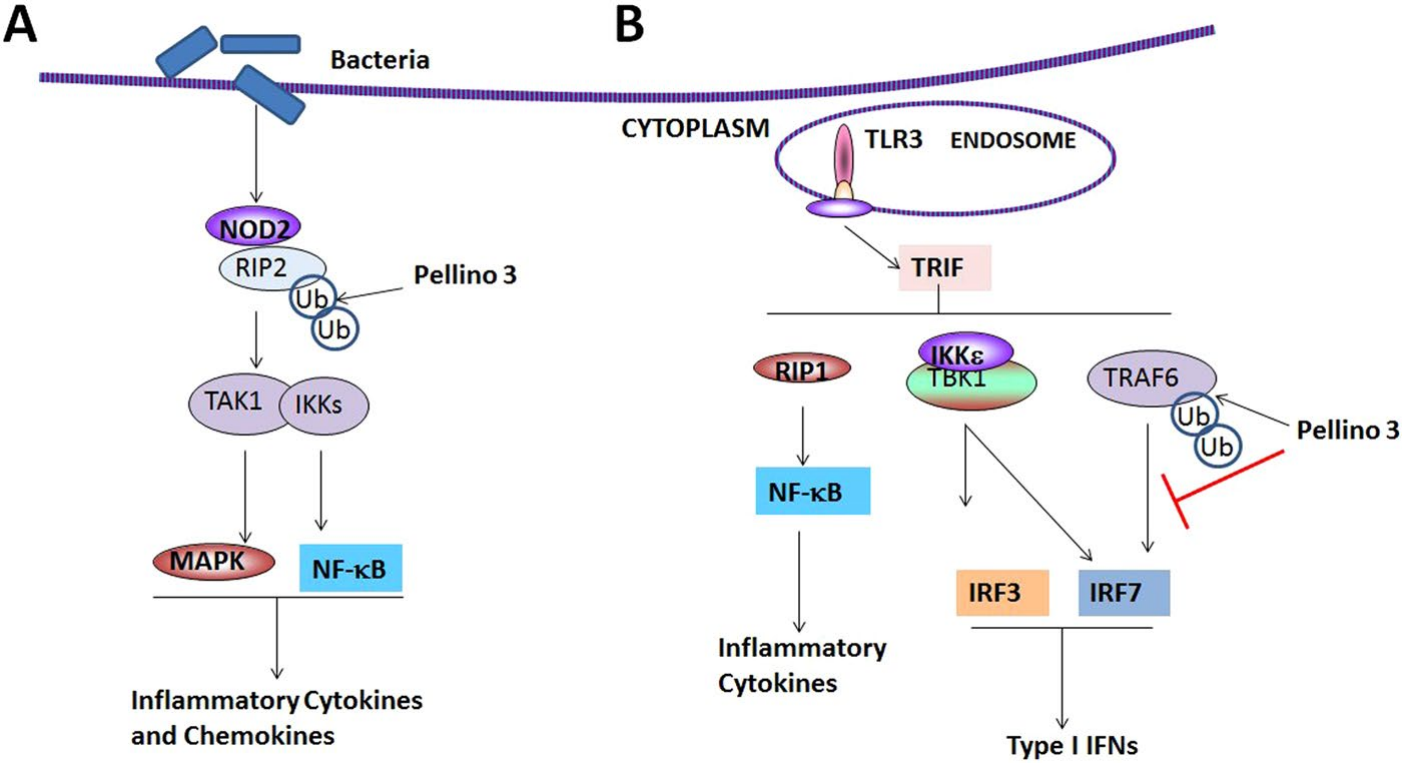
Schematic demonstrating the role of Pellino 3 in inflammation. (**A**) Recognition of bacterial products by nucleotide-binding oligomerization domain-containing protein 2 (NOD2), results in NOD2 oligomerization and recruitment of receptor-interacting-serine/threonine-protein kinase 2 (RIP2). This complex then recruits Pellino 3 which ubiquitinylates (Ub) RIP2 leading to the recruitment of TGFβ-activated kinase 1 (TAK1) and the IKK complex. This facilitates activation of mitogen-activated protein kinase (MAPK) and nuclear factor-κB (NF-κB) pathways culminating in the induction of inflammatory cytokines and chemokines. (**B**) Ligand interactions with Toll-like receptor 3 (TLR3) results in the recruitment of TIR domain-containing adaptor protein-inducing IFNβ (TRIF) which initiates a signal cascade that results in the phosphorylation and ubiquitylation of interferon-regulatory factor 3 (IRF3) and IRF7 via TANK-binding kinase 1 (TBK1)–IκB kinase-ε (IKKε)and TNF receptor-associated factor 6 (TRAF6) respectively. Nuclear translocation of IRF3 and IRF7 induce the expression of type I IFNs. Additionally the TBK1–IKKε–IRF3 pathway also induces the Pellino 3 expression resulting in ubiquitylation of TRAF6 and inhibition of type I IFN expression.

PELI3, a member of the Pellino E3 ubiquitin ligase family, is a known regulator of inflammation via interactions with Toll-like receptors^32^. PELI3 is expressed in most tissues, and has been reported to interact with IRAK1, NF-κB-inducing kinase, TRAF6, and transforming growth factor-β activated kinase 1 (TAK1)^56^. Its contributes to control of the innate immune system through pathways downstream from TLR-3 activation by regulating the expression of Type 1 interferons (T1IFN)^57^, nucleotide-binding oligomerization domain-containing protein 2 (NOD2) activation of NF-κB^58^ and the TNF-induced activation of NF-κB^59^ (Fig. 4). Other autoimmune conditions have been linked to PELI3 dysfunction, including inflammatory bowel disease^60^ and multiple sclerosis (MS)^61^. Murine studies using PELI3 knockout mouse have shown increased levels of T1IFNs in response to TLR3 stimulation with Poly(I:C)^33^. In the context of ocular inflammation PELI3 represents an interesting target as it functions as a negative regulator of T1IFN^58^ and pSS has been characterized by T1IFN signature in the salivary glands and systemically^62^.

Both gain and loss of function studies demonstrate that transfecting PECs with miR-744-5p mimic or antagomir has reciprocal effects on PELI3 expression and analysis of the ability of miR-744-5p mimic to bind the 3′ UTR of PELI3 confirms PELI3 as a direct target. Previous reports have suggested that reduced PELI3 expression contributes to disease pathogenesis via its role in regulating inflammation^60,61^. In keeping with this we have shown that increasing PELI3 expression via use of a miR-744-5p antagomir in PECs under conditions that mimic pSS inflammation results in significant reductions in IFN dependent chemokines Rantes and CXCL10. Which is significant given the role of inflammatory cytokines and chemokines to disease induction in mouse models of pSS^42,43^.

Taken together our results demonstrate a novel role for PELI3 in the regulation of ocular surface inflammation and suggest that modulation of its expression using a miR-744-5p antagomir has potential therapeutic implications.

## Materials and methods

### Ethics

This study was reviewed and approved by the Research and Ethics Committee of the Royal Victoria Eye and Ear Hospital (RF2012) and written informed consent was obtained from all participants. This study was conducted in accordance with the Helsinki Declaration and applicable regulations.

### Patient recruitment

All pSS patients were recruited from the Royal Victoria Eye and Ear Hospital, Adelaide Road, Dublin 2, Ireland. Thirty consecutive patients who were previously diagnosed with pSS or suspected of having pSS were thoroughly assessed using clinical history, clinical examination, slit lamp examination and dry eye tests. Of these, 20 patients were confirmed to have pSS based on the American European Consensus Group (AECG) Classification Criteria for Sjögren’s syndrome^63^. Those who did not satisfy the AECG criteria were excluded from the subsequent analysis. The mean age of the patients was 57.9 years (range 35-70 years) with an approximately 4:1 ratio of females to males. All patients had had sicca symptoms with dry eyes and dry mouth while 75% of them demonstrated systemic manifestation which is consistent with other studies^2,64^. Patient demographics and results of ocular surface parameters are summarised in Table 1. Clinical data and medication history were recorded for each patient at the time of the blood draw and summarised in Supplemental Tables 1 and 2 respectively. Age- and sex-matched healthy donors who had no history of ocular or autoimmune diseases or treatment with immunosuppressive agents were also recruited as controls.

### Ocular wash collection and analysis

To collect ocular wash samples, 30 μL of phosphate-buffered saline (PBS) was instilled into the inferior fornix (without topical anesthetics). Tear fluid and buffer were collected with a micropipette and placed into a 1.5-mL Eppendorf tube and stored at −80 C until further examination. Ocular washes from pSS patients and healthy controls were analysed using a multiplex cytokine ELISA from Meso Scale Discovery (MSD) which included interferon gamma (IFNγ), interleukin (IL)-10, IL-12p70, IL-13, tumour necrosis factor alpha (TNFα), IL-2, IL-4, IL-5, IL-8 and interleukin 1beta (IL-1β).

### Isolation of primary human conjunctival cells by impression cytology

Impression cytology was preformed using a Biopore membrane (Merck Millipore, Darmstadt Germany) as previously decribed^65^. Briefly topical Minims Proxymetacaine hydrochloride 0.5% (Bausch & Lomb, Surrey United Kingdom) was instilled into the inferior fornix of both eyes. A Barraquer speculum was then inserted. Air drying was performed for 15 seconds prior to testing 6 areas of the bulbar conjunctiva being sampled, taking care to avoid the fornix. The membrane was then applied gently but firmly against the conjunctiva, with the rim of the cylinder just adjacent to the limbus, for 15 seconds. This prevents the membrane from touching the limbus and collecting limbal material. This procedure was repeated for both eyes. For gene and miR expression studies all 12 membranes were processed in TRI Reagent^®^ (Sigma). For transfection studies cells were dissociated from the membrane following incubation with trypsin/EDTA (Labtech). Cells were visualised using an inverted bright field microscope (Nikon Eclipse TS100) x 10 magnification.

### miRNA expression profile assay

A microarray screen of miR expression in the primary human conjunctival epithelial cells (PECs) from pSS patients (n = 5) and healthy controls (n = 5) was performed with the use of ORB MirBASE Version 19 MicroRNA Microarray, Ocean Ridge Bioscience (Palm Beach Gardens, FL). Quality control of the total RNA samples was assessed using UV spectrophotometry. The samples sent to Ocean Ridge Biosciences were checked for quality by Bioanalyzer on Agilent 2100 Bioanalyzer RNA 6000 Pico Chip(s). The RIN numbers were used to determine whether or not the samples were intact, partially/moderately degraded, degraded or ultimately degraded. For miR data, log2 transformed probe intensities were normalized by subtracting the normalization factor (N = 20% trim mean of the non-saturated human probes above threshold in all samples) and scaled by adding the grand mean of the normalization factor across all samples. The As for mRNA, the log2 transformed probe intensities were normalized by subtracting the 70^th^ percentile of the human probes (for each sample) and scaled by adding the grand mean of the 70^th^ percentile of the human probes across all samples. The criteria for detection of the miR and mRNA is that the signal for a given miR and mRNA must be above the normalized thresholds in >25% of the samples. Correction for multiple testing was performed using Paired T-Test P values and the adjusted P values that controls the False Discovery Rate (FDR). The P values were adjusted according to the method of Benjamini and Hochberg^66^.

### Real-time polymerase chain reaction (qPCR)

RNAs were extracted from conjunctival epithelial cells using TRI Reagent® (Sigma). Samples were reverse transcribed to complementary DNA using Tetro cDNA Synthesis Kit (Bioloine) or miRScript II RT kit (Qiagen) according to the manufacturer’s recommendations for gene and miR analysis respectively. Real-time quantitative PCR investigating gene expression was performed using primer sequences in Table 2 with SYBR Green Taq ReadyMix (Sigma) as per manufacturer’s recommendations. Data were analyzed using an ABI Prism 7900 system (Applied Biosystems). Genes were normalised to an RNU6B reference. miRs were normalised to the U6 small nuclear RNA (U6 snRNA). Real-time PCR data were analyzed using the 2-ddct method^67^.

**Table 2:** Real-time quantitative PCR primer sequences.

Gene/miR	Forward primer	Reverse Primer
miR-744-5p	AAGGTGCGGGGCTAGGGCTAA	AGTAAGGTTGAGGTTA
Pellino3	GATGGCCTGATGGATGGACTG	AGGTCGATGAGAGAGCCGTC
Rantes (CCL5)	CCTCGCTGTCATCCTCATTGCT	TACTCCCGAACCCATTTCTTCTC
CXCL10	GGAAGCACTGCATCGATTTTG	CAGAATCGAAGGCCATCAAGA
CK18	GGCATCCAGAACGAGAAGGAG	ATTGTCCACAGTATTTGCGAAGA
CK19	ACCAAGTTTGAGACGGAACAG	CCCTCAGCGTACTGATTTCCT
RNU6B	CTCGCTTCGGCAGCACA	AACGCTTCACGAATTTGCGT
18 s	GGGAGGTAGTGACGAAAAAT	ACCAACAAAATAGAACCGCG

### Mimic and inhibitor transfection

MicroRNA-744-5p oligonucleotides were obtained from Exiqon. Negative controls were based on the sequences of miRNA in *Caenorhabditis elegans* (cel-miR-67). Cells were seeded at 1 × 10^5^ cells/well in a 96-well for primary human conjunctival epithelial cells and HConECs. Cells were then transfected with 50 nM of either a negative control/miR-744-5p mimic or antagomir. Transfection of primary human conjunctival cells and HConEC was performed using Metafectene SI transfection reagent as recommended by the manufacturer’s protocol. Evaluation of the experiment was carried out 72 h after transfection.

### Luciferase reporter assay

The putative miR-744-5p target sequence in the 3′UTR of human Pellino3 (site 1) and an unrelated fragment control region of the 3′UTR of human Pellino3 (site 2) was cloned into the psiCHECK-2 vector (Promega) downstream of the *Renilla* luciferase reporter gene with the primers sequences in Table 3. All constructs were sequenced, and were prepared with the use of an EndoFree Plasmid Maxi kit (Qiagen). 293 T cells were seeded at 1×10^5^ cells/well in a 96-well plate 1-day pre-transfection and then transfected with a mixture of 50 ng Pellino3-UTR site 1 or site 2 luciferase reporter vector and 50 nM of either a negative control/miR-744-5p mimic. The cells were harvested 48 h later, and luciferase activity was assessed using a Dual Luciferase Reporter Assay System (Promega). Firefly luciferase was used to normalise the *Renilla* luciferase. All experiments were carried out in triplicate.

**Table 3:** Luciferase reporter primers sequences.

Name	Sequence: (5′ to 3′)
Site 1 FP	GAGAGACTCGAGACCTCGCTGCTCAGCTGCCC
Site 1 RP	GAGAGAGCGGCCGCTCTGGAGAGTGCTCAATGGA
Site 2 FP	GAGAGACTCGAGAGTTCACAGTCTAGTGGAGG
Site 2 RP	GAGAGAGCGGCCGCTCCACAAATGAGGTTCAGAA

### Culture of primary human conjunctival epithelial cells (HConEC)

Primary human conjunctival epithelial cells (HConEC) were purchased from Innoprot. These cells are isolated from human conjunctiva, cryopreserved at primary culture and guaranteed to further expand for 15 population doublings at the conditions provided in the data sheet. HConECs were cultured in Corneal Epithelial Cell Medium (CEpiCM) which is designed for optimal growth of normal human corneal epithelial cells *in vitro* and contains essential and non-essential amino acids, vitamins, organic and inorganic compounds, hormones, growth factors and trace minerals. For some experiments HConECs were either left untreated or treated with polyinosinic:polycytidylic acid (Poly(I:C)) 20 µg/ml during the final 24 hours of culture. Transfection of FITC-labelled miR-744-5p antaogmir (Exiqon) was visualised using an inverted bright field microscope (Leica, DMIL) x20 magnification. Gene expression was investigated by real-time quantitative PCR and production of Rantes (CCL5) and CXCL10 was determined by ELISA (RnD Systems). HConECs were mycoplasma free and characterised by expression of CK18, CK19 by PCR.

### Flow cytometry

Single-cell suspensions of HConECs were prepared 72 h after transfection with a FITC labelled miR-744-5p antagomir (Exiqon). Briefly, cells were suspended in fluorescence-activated cell sorting (FACS) buffer containing 1×phosphate-buffered saline (PBS) (pH 7.2), 2% fetal bovine serum (Sigma), and 0.1% sodium azide. 10,000 events were acquired on a BD Biosciences FACS Canto II. Data analysis was performed by using FlowJo_V10 (Tree Star). Negative antagomir treated cells were used as negative controls.

### Statistical analysis

Data were analyzed using Prism 6 software, version 6.07 (GraphPad Software, La Jolla, CA, USA). The nonparametric Mann-Whitney test was used to compare differences in gene expression, miR expression and cytokine levels between patients and controls. The Students paired t test was performed to examine differences in miR, protein and cytokine levels between transfected cells and Poly(I:C) treated cells. Data deemed significantly different at *P* values less than 0.05.

## Supplementary information

Supplemental file.
