# Supplementary material for: miR-744-5p contributes to ocular inflammation in patients with primary Sjogrens Syndrome
Source: Sci Rep. 2020 May 4;10:7484. doi: 10.1038/s41598-020-64422-5 (PMC7198540; doi:10.1038/s41598-020-64422-5)
Supplement: Supplementary file 1 — Supplemental file. [file 41598_2020_64422_MOESM1_ESM.docx]

**miR-744-5p contributes to ocular inflammation in patients with primary Sjogrens Syndrome**

Qistina Pilson, MB Bch BAO MRCSI FEBO (2,3), Siobhan Smith, PhD (1), Caroline A. Jefferies PhD (4,5), Joan Ní Gabhann-Dromgoole*, PhD (1, 2) and Conor C. Murphy*, PhD FRCSI (2,3).

*Authors contributed equally

(1) School of Pharmacy and Biomolecular Sciences (PBS) and RSCI Research Institute, Royal College of Surgeons in Ireland, Dublin 2, Ireland.

(2) Department of Ophthalmology, Royal College of Surgeons in Ireland, Dublin 2, Ireland.

(3) Department of Ophthalmology, Royal Victoria Eye and Ear Hospital, Dublin 2, Ireland.

(4) Division of Rheumatology, Department of Medicine, Cedars-Sinai Medical Centre, 8700 Beverly Blvd, Los Angeles, California 90048, USA.

(5) Department of Biomedical Sciences, Cedars-Sinai Medical Centre, 8700 Beverly Blvd, Los Angeles, California 90048, USA.

Please address correspondence to Professor Conor Murphy, **RCSI** Department of Ophthalmology, Royal College of Surgeons in Ireland, Royal Victoria Eye and Ear Hospital, Adelaide Road, Dublin 2, Ireland.

Tel: 353 1 4022226. Email: [conorcmurphy@rcsi.ie](mailto:conorcmurphy@rcsi.ie)


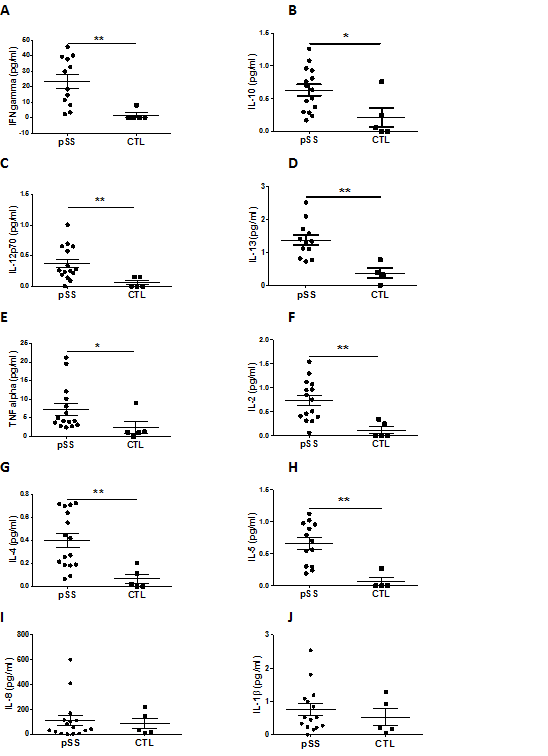


**Supplemental Figure 1.** **Cytokine levels from ocular washes of pSS patients and healthy controls.** Ocular washes were collected and levels of indicated cytokines were determined by multiplex ELISA (MSD). Graphs show concentration (pg/ml), *p<0.05 and **p<0.01 were considered statistically significant.

Supplemental Table 1: List of pSS patients past and current medications for the management of glandular and extra glandular manifestations

| **Medications** | **Total (%)**  **n=20** | **Current (%)** | | **Past (%)** | |
| --- | --- | --- | --- | --- | --- |
|  |  |  | |  | |
| **Hydroxychloroquine** | 9 (45) | 7 (28) | | 2 (10) | |
| **Systemic Prednisolone** | 3 (15) | 0 | | 3 (15) | |
| **Pilocarpine** | 5 (25) | 3 (15) | | 2 (10) | |
| **Ocular lubricants** | 20 (100) | 20 (100) | | 20 (100) | |
| **Oral lubricants** | 5 (25) | 5 (25) | | 0 | |
| **Methotrexate** | 1 (5) | 0 | | 1 (5) | |
| **Rituximab** | 1 (5) | 1 (5) | | 0 | |
| **Azathioprine** | 1 (5) | 1 (5) | | 0 | |
| **Mycophenolate mofetil** | 1 (5) | 0 | | 1 (5) | |
|  |  | |  | |  |

Supplemental Table 2: Blood test results for pSS patients. Data shown below are the number and percentage of patients who were positive for the blood tests when categorized under AECG criteria.

| **Blood tests (Abnormal)** | **AECG**  ***N*=20 (%)** |  |
| --- | --- | --- |
|  |  |  |
| **Lymphopenia** (≤1.0 e9/L) | 5/20 (25) |  |
| **Neutropenia** (≤1.55 e9/L) | 1/20 (5) |  |
| **Anaemia** (<13.5 g/dL) | 5/20 (25) |  |
| **Thrombocytopenia** (<164 e9/L) | 0/20 (2) |  |
| **ANA+** (>1.0 ratio) | 19/20 (95) |  |
| **Rheumatoid factor+** (>30 IU/mL ) | 14/20 (70) |  |
| **Anti Ro/SSA+** (≥7.0 ELIA U/mL) | 18/20 (90) |  |
| **Anti La/SSB+** (≥7.0 ELIA U/mL) | 13/20 (65) |  |
| **Anti DS DNA+** (>10.0 ELIA U/mL) | 2/20 (10) |  |
| **Anti sm+** (≥7.0 ELIA U/mL) | 0/20 (0) |  |
| **Anti Jo-1+** (≥7.0 ELIA U/mL) | 0/20 (0) |  |
| **Anti Scl-70+** (≥7.0 ELIA U/mL) | 0/20 (0) |  |
| **Anti MPO+** (≥2.0. IU/mL) | 0/20 (0) |  |
| **Anti PR3+** (≥ 3.5 IU/mL ) | 0/20 (0) |  |
| **Monoclonal gammopathy** | 0/20 (0) |  |
| **Low complement C3** (<0.82 g/L) | 2/20 (10) |  |
| **Low complement C4** (<0.15 g/L) | 5/20 (25) |  |
| **Thyroid function tests** ^ε^ | 0/20 (0) |  |
|  |  |  |
|  |  |  |

^ε^ Thyroid function tests (includes free T4 and TSH)
